# Quantitative analyses of factors related to anxiety and depression in patients with retinitis pigmentosa

**DOI:** 10.1371/journal.pone.0195983

**Published:** 2018-04-23

**Authors:** Mayumi Sainohira, Takehiro Yamashita, Hiroto Terasaki, Shozo Sonoda, Kazunori Miyata, Yusuke Murakami, Yasuhiro Ikeda, Takeshi Morimoto, Takao Endo, Takashi Fujikado, Junko Kamo, Taiji Sakamoto

**Affiliations:** 1 Department of Ophthalmology, Kagoshima University Graduate School of Medical and Dental Sciences, Kagoshima, Japan; 2 Miyata Eye Hospital, Miyazaki, Japan; 3 Department of Ophthalmology, Graduate School of Medical Sciences, Kyushu University, Fukuoka, Japan; 4 Department of Applied Visual Science, Osaka University Graduate School of Medicine, Osaka, Japan; 5 Department of Ophthalmology, Osaka University Graduate School of Medicine, Osaka, Japan; 6 Department of Ophthalmology, Kofu Kyoritsu Hospital, Yamanashi, Japan; Massachusetts Eye & Ear Infirmary, Harvard Medical School, UNITED STATES

## Abstract

The purpose of this study is to determine the factors related to anxiety and depression in patients with retinitis pigmentosa (RP). The status of anxiety and depression was determined in RP patients with the Hospital Anxiety and Depression Scale (HADS) questionnaire which consisted of subscales for HADS-anxiety (HADS-A) and HADS-depression (HADS-D). The vision-specific quality of life (VSQOL) was assessed with the National Eye Institute Visual Function Questionnaire 25 (NEI-VFQ25). The correlations between the HADS-A or HADS-D scores and vision-related clinical parameters such as the best-corrected visual acuity (BCVA), Functional Acuity Score, Functional Field Score, Functional Vision Score, the NEI- VFQ25 subscale score were determined. The socioeconomic status, such as the work status and membership in the RP society, was investigated to determine the factors related to the HADS-A and HADS-D scores. One hundred and twelve RP patients (46 men and 66 women) with mean age of 60.7±15.4 (standard deviation) years were studied. The HADS-A score was not significantly correlated with any visual functions but was significantly correlated with the general health condition (r = -0.34, *P*<0.001) and the role limitation (r = -0.20, *P* = 0.03) of the NEI-VFQ25 subscale. The HADS-D score was significantly correlated with all the visual functions (r = -0.38 to 0.29, *P*<0.001), the NEI-VFQ25 subscale score (r = - 0.58 to -0.33, *P*<0.001) by Spearman’s correlations. The HADS-A score was significantly higher in the members of the RP society than in non-members (*P* = 0.013). The mean HADS-D score of employed individuals was significantly lower than that of unemployed ones (*P* = 0.001) by the Mann-Whitney U test. The results indicate that visual function impairments and vision-related quality of life are associated with a depressive state, and the general health condition is related to anxiety in RP patients. Being employed may be strongly correlated with the degree of depression in RP patients.

## Introduction

Retinitis pigmentosa (RP) is an inherited, progressive ocular disease that is associated with degeneration of the photoreceptors. It is estimated that 2.0 million individuals are affected with RP worldwide [1]. Although much effort has been made in developing new treatments, there is no effective treatment for RP [2–8]. Because RP is a progressive disease and can lead to severe visual impairments, it can lead to severe psychological distress. The lack of effective therapy and the deteriorating nature of RP causes a continuous psychological burden on patients throughout their life. Indeed, many earlier studies have reported that visual impairments are risk factors for anxiety and depression in RP patients [9–16]. Visual impairments are also strongly associated with social issues such as employment and education. It has been reported that patients with RP have lower educational levels, lower employment rates, and lower income levels [17]. A recent report from France supported the idea that RP was closely related to social difficulties which can then lead further mental health problems especially in younger patients [16]. To ameliorate these problems and to reduce the suffering of patients with RP, it is indispensable to know the factors that cause the mental health problems.

Thus, the purpose of this study was to determine the factors related to the anxiety and depression in RP patients. To accomplish this, we used the Hospital Anxiety and Depression Scale (HADS) questionnaire to determine the factors significantly correlated with the mental health of Japanese RP patients.

## Materials and methods

### Ethics statement

All of the procedures used in this study conformed to the tenets of the Declaration of Helsinki, and this study was approved by the Ethics Committee of Kagoshima University Hospital (Kagoshima, Japan), and Miyata Eye Hospital (Miyazaki, Japan), Kofu Kyoritsu Hospital (Yamanashi, Japan), Osaka University Hospital (Osaka, Japan), and Kyushu University Hospital (Fukuoka, Japan). This study was registered with the University Hospital Medical Network (UMIN) with the title, Anxiety and depression in patients with retinitis pigmentosa (No. UMIN000018444).

### Subjects

This was a prospective, cross-sectional study. All of the patients were consecutively asked to participant in this study at Kagoshima University Hospital, Miyata Eye Hospital, Kofu Kyoritsu Hospital, Osaka University Hospital, and Kyushu University Hospital during August 2015 to February 2017. When a patient declined to participate, the patient was excluded. Thus, this can lead to a potential bias but it was minimized by the recruiting cases consecutively.

All patients had bilateral RP and were diagnosed by ophthalmic examinations including slit-lamp biomicroscopy, dilated funduscopy, electroretinography (ERG; Tomey LE-4000, Tokyo, Japan), optical coherence tomography (OCT, Heidelberg Spectralis, Heidelberg, Germany), and measurements of the best-corrected visual acuity (BCVA) in decimal units. The retinitis pigmentosa was diagnosed by the presence of nyctalopia, constricted visual fields, and characteristic fundus findings of arteriolar narrowing, fine intraretinal pigmentation, and loss of retinal pigment epithelium (RPE) typically in the mid- and far periphery. Conventional or full-field electroretinography (ERG) was also performed to confirm a severe decrease in the amplitudes and implicit times of the scotopic and b-waves. Optical coherence tomography (OCT) was performed to assess the vitreoretinal interface, retinal configuration, presence of intraretinal or subretinal fluid, photoreceptor layer, and RPE status. These findings were used to exclude 7 of 119 eyes with non-RP disorders such as vitreomacular traction. Patients with non-RP ocular diseases that could cause visual impairments were also excluded. All participants who had a history of psychiatric treatment or were using psychotropic drug were excluded.

## Procedures

The state of anxiety and depression was determined by the answers to the Japanese version of the HADS questionnaire. The HADS was originally developed by Zigmond and Snaith in 1983 [18,19], and it has been used to determine the anxiety and depression levels in individuals with physical health problems [20–22].

The HADS questionnaire is composed of two subscales, viz., the HADS-anxiety, (HADS-A) and HADS-depression (HADS-D) scales. This questionnaire was developed by experts in the field of mental health to identify anxiety and depression [22]. The HADS questionnaire has been validated by more than 70 studies and more than 1400 reports [23]. Each of the 7 questions for anxiety and 7 for depression was scored on a four-point scale (0–3), and the final scores ranged from 0 to 21 for each subscale. Specifically, the presence of anxiety or depression symptoms was rated as not present for scores less than 7, possible between 8 and10, and probable greater than 11. The cutoff values of HADS were determined from the earlier report [22].

The vision-specific quality of life was determined by the National Eye Institute Visual Function Questionnaire 25 (NEI-VFQ 25) [23,24]. The NEI-VFQ 25 questionnaire was developed to determine the vision-related quality of life of patients with chronic eye diseases. We used the Japanese version of the NEI-VFQ 25 [25]. This questionnaire has 25 questions for 12 vision-associated aspects of life; general health, general vision, ocular pain, near vision, distant vision, social function, role limitation, dependency, driving, color vision, peripheral vision, and mental health. The subscale scores ranged from 0 to 100, and higher scores indicated a better QOL with less impairments. We excluded ocular pain and driving because previous studies have reported that ocular pain was not directly related to the visual conditions, and the driving because all subjects had already stopped driving because of their visual conditions [26, 27].

The best-corrected visual acuity (BCVA) was determined with the Snellen Visual Acuity Charts, and the decimal BCVA was converted to a logarithm of the minimum angle of resolution (logMAR) units for the statistical analyses. The visual fields were determined by Goldmann perimetry using the III/4/e isopter. The Functional Vision Score (FVS) was calculated using the Functional Acuity Score (FAS) and Functional Field Score (FFS) based on the American Medical Association guides for the analysis [28].

The aspects of the socioeconomic status were examined by the following background questions. The time after the diagnosis of RP by >1 year or ≤1 year, history of serious systemic diseases including myocardial infarction, cerebral infarction, Parkinson's disease, and cancer by Yes or No, living alone by Yes or No, presence of a person to consult by Yes or No, death of close person within 1 year by Yes or No, job status by employed or unemployed, experience with vison rehabilitation service by Yes or No, and membership in Japan RP Society by Yes or No.

## Statistical analyses

The data were analyzed with the SPSS Statistics 21.0.0.1 software as a statistical plan to investigate factors related to HADS-A or HADS-D. Spearman’s correlation analysis was used to determine the significance of the correlations between the HADS-A or HADS-D score and the BCVA of the better eye, of the worse eye, the FAS, FFS, FVS, the NEI-VFQ25 subscale scores, and the mean score. The Mann-Whitney U test was used to analyses the effects of the socioeconomic status in HADS-A or HADS-D. The statistical significance level was set at <0.05.

## Results

One hundred twelve patients of 119 (94%) completed the HADS and the NEI-VFQ25 questionnaires. The demographic and clinical characteristics of these subjects are summarized in Table 1.

**Table 1:** Demographics and clinical characteristics of participants.

	RP patients
Number	112
Age (years)	60.7 ± 15.4 (20–90)
Sex (men/women)	46/66
BCVA (logMAR units)	
Better eye	0.61 ± 0.74 (-0.18–2.8)
Worse eye	0.94 ± 0.93 (-0.08–2.9)
HADS-A subscore	6.4 ± 3.8
HADS-D subscore	6.1 ± 3.6
FAS	68.1
FFS	40.7
FVS	32.7
Mean NEI-VFQ25	49.0 ± 20.1

RP, retinitis pigmentosa; logMAR, logarithm of the minimum angle of resolution; BCVA, best-corrected visual acuity; HADS-A, Hospital Anxiety and Depression Scale-Anxiety; HADS-D, Hospital Anxiety and Depression Scale-Depression; FAS, Functional Acuity Score; FFS, Functional Field Score; FVS, Functional Vision Score; NEI-VFQ25, National Eye Institute Visual Function Questionnaire—25 items. subscale Data are the means ± standard deviations.

**Fig 1:**
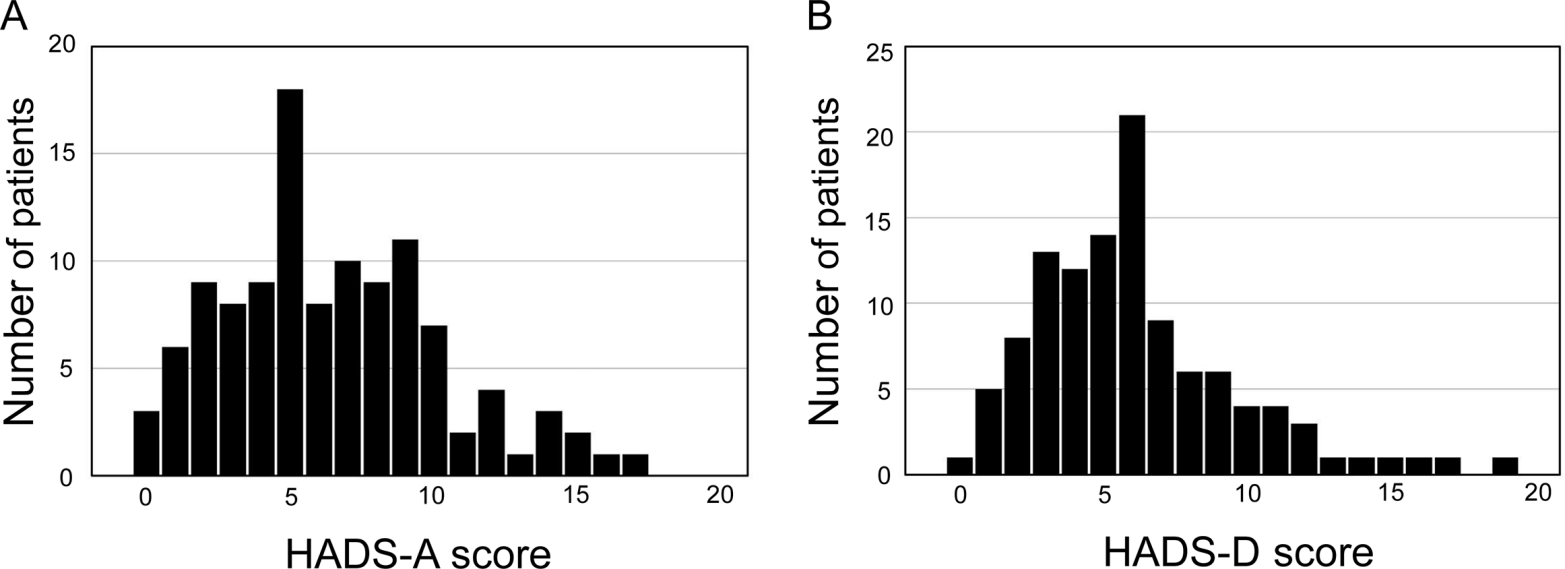
Distribution of HADS scores. Score definition. Normal, 0–7; possible, 8–10; probable: 11–21. HADS: Hospital Anxiety and Depression Scale, HADS-A, HADS anxiety subscale; HADS-D, HADS depression subscale. A. Distribution of HADS-A score. B. Distribution of HADS-D scores. Anxiety was present in 37% (41/112) and depression in 26% (29/112) of the RP patients.

The mean of the HADS-A score was 6.4 ± 3.8 (standard deviation, Fig 1A), the HADS-D score was 6.1 ± 3.6 (Fig 1B), and the mean NEI-VFQ25 score was 49.0 ± 20.1 in the RP patients.

**Fig 2:**
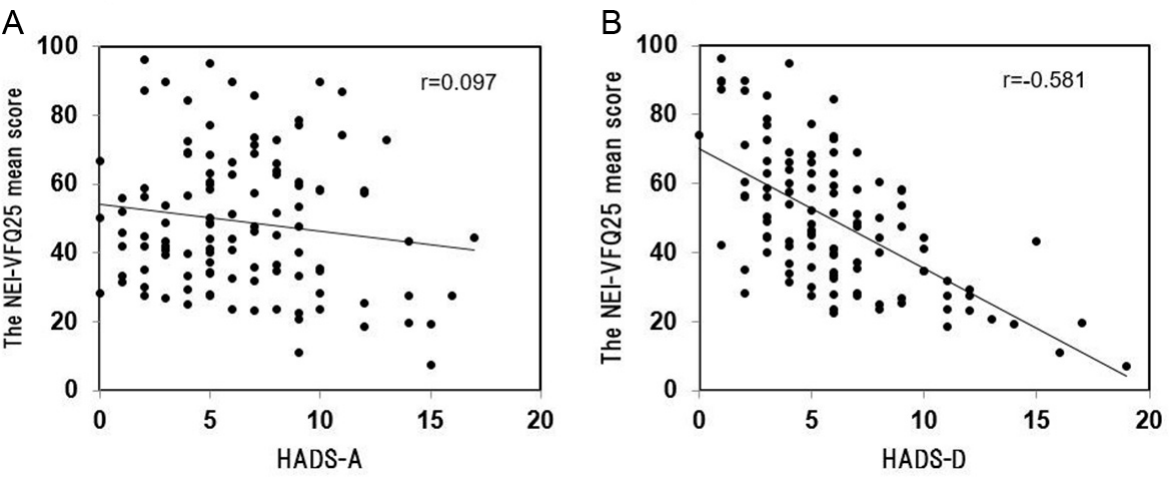
Correlation between the HADS score and the NEI-VFQ25 mean score in patients with retinitis pigmentosa. 2A. Correlation between the HADS-A score and the NEI-VFQ25 mean score, Spearman's rank correlation coefficient (r = - 0.097, *P* = 0.31). 2B. Correlation between the HADS-D score and the NEI-VFQ25 mean score (r = - 0.581, *P* <0.01).

The HADS-A score was not significantly correlated with the BCVA of the better eye (r = -0.09, *P* = 0.35) or the worse eye (r = -0.11, *P* = 0.26). The other visual functions such as the FAS (r = 0.11, *P* = 0.27), FFS (r = 0.12, *P* = 0.20), and FVS (r = 0.09, *P* = 0.34) were also not significantly correlated with the HADS-A score. There was no significant correlation between HADS-A score and the mean score of NEI-VFQ 25 (r = -0.097, *P* = 0.31) (Fig 2A). However, the general health condition (r = -0.34, *P* <0.01) and the role limitations (r = -0.20, *P* = 0.03) of the NEI-VFQ 25 subscale score were significantly correlated with the HADS-A score (Table 2). In addition, the HADS-D score was correlated significantly with the BCVA of the better eye (r = 0.29, *P* < 0.01) and of the fellow eye (r = 0.28, *P* < 0.01), FAS (r = -0.30, *P* = 0.002), FFS (r = -0.32, *P* <0.01), FVS (r = -0.38, *P* <0.01), all of the NEI-VFQ25 subscale score (r = -0.58 to -0.33, *P* <0.01), and the mean score (r = -0.58, *P* <0.01; Fig 2B; Table 2).

**Table 2:** Sperman's correlation between the HADS-A or HADS-D score and variables in patients with retinitis pigmentosa.

	HADS-A		HADS-D	
Variables	r	P value	r	P value
HADS-A			0.48	<0.01
HADS-D	0.48	<0.01		
BCVA (logMAR)				
Better eye	-0.09	0.35	0.29	<0.01
Worse eye	-0.11	0.26	0.28	<0.01
FAS	0.11	0.27	-0.30	<0.01
FFS	0.12	0.20	-0.32	<0.01
FVS	0.09	0.34	-0.38	<0.01
NEI-VFQ-25 (total score)	-0.10	0.31	-0.38	<0.01
General health	-0.34	<0.01	-0.53	<0.01
General vision	0.01	0.90	-0.33	<0.01
Near vision	0.00	0.98	-0.39	<0.01
Distant vision	-0.07	0.49	-0.47	<0.01
Social function	0.01	0.94	-0.47	<0.01
Role limitation	-0.20	0.03	-0.58	<0.01
Dependency	-0.17	0.08	-0.58	<0.01
Color vision	-0.11	0.27	-0.52	<0.01
Peripheral vision	0.03	0.74	-0.33	<0.01
Mental health	-0.15	0.11	-0.52	<0.01

HADS-A, Hospital Anxiety and Depression Scale-Anxiety; HADS-D: Hospital Anxiety and Depression Scale-Depression; BCVA: best-corrected visual acuity; logMAR: logarithm of the minimal angle of resolution; FAS: Functional Acuity Score, FFS: Functional Field Score, FVS:Functional Vision Score; NEI-VFQ 25: National Eye Institute Visual Function Questionnaire 25 items

No significant differences were observed between Vision rehabilitation, Social support, Death of close person, Living style, Serious disease, and Diagnosis. However, the HADS-A score of the members of the RP society was significantly higher than that of the non-members (*P* = 0.013). The HADS-D score of employed individuals was significantly lower than that of unemployed ones (*P* = 0.001) by the Mann-Whitney U test. (Table 3).

**Table 3:** The difference of socioeconomics status in the HADS-A or HADS-D score by Mann Whitney test.

	HADS-A	HADS-D
Socioeconomic status	P value	P value
JRPS member	0.013	0.33
Vision rehabilitation	0.47	0.07
Employment	0.29	0.001
Social support	0.98	0.40
Death of close person	0.99	0.72
Living style	0.77	0.67
Serious disease	0.17	0.86
Diagnosis	0.70	0.07

HADS-A: Hospital Anxiety and Depression Scale-Anxiety, HADS-D:Hospital Anxiety and Depression Scale-Depression

## Discussion

Our results showed that the percentage of RP patients with anxiety was 37% and that of depression was 26%. Chaumet-Riffaud et al reported that 36.5% of RP patients had anxiety and 15.5% had depression using the HADS questionnaire [16]. Hahm et al reported that 25.7% of RP patients had depression using the Beck Depression Inventory (BDI) [12]. The percentage of RP patients with anxiety in our cohort was comparable to these two earlier studies but the percentage of depression was far higher [12,16]. This difference might be because the average age of their subjects was 30 years which was much younger than the mean age of our cohort at 60.7 ± 15.4 years.

It is important to note that the depressive state was found to be significantly associated with every aspect of visual function including the BCVA in the better or worse eye, and the FAS, FFS, FVS and the NEI-VFQ25 subscale score. These significant correlations were also found in other studies [12–15]. Thus, Hahm et al found a significant relationship between visual functions and a depressive state in RP patients, however it was not found in such visual functional tests as the weighted visual acuity score or FVS [12]. In their study, the psychological state was evaluated by the Beck Depression Inventory (BDI), and their study group was younger than our group. This difference might account for the differences.

Our results showed that the RP patients who were employed had significantly lower depression scores than those who were unemployed. In a study of young RP patients in France, employment was found to be significantly associated with a lower incidence of depression [16]. It has been established that financial independence is an important factor for a stable psychological state, and this most likely accounts for the significant relationship between employment and the depressive state of the RP patients. In addition, having good vision is an important factor in keeping a job. These findings indicate that doctors who treat RP patients need to treat not only the ocular problems but also the social problems such as employment and financial security.

The degree of anxiety of RP patients was significantly correlated with the general health or the role limitation of the NEI-VFQ25 subscales but not with any visual functions. Chaumet-Riffaud et al also reported that anxiety was not significantly correlated with the visual functions although the depressive state tended to worsen as the visual functions decreased [16]. It has been reported that anxiety was not significantly associated with visual functions in patients with age-related macular degeneration [29]. Thus, the absence of a significant relationship between anxiety and visual functions might be a universal psychological reaction to ocular diseases.

A significant correlation was found between the anxiety score and depression score (r = 0.48; *P <*0.01) among all patients. Because having good vision is fundamental for a reasonable private and social life, it is understandable that those with poor vision can have anxiety. In RP, the progression of the disease is slow, and thus RP may not cause serious anxiety if the patient understands the nature of RP accurately. However, if an additional factor is present, it can enhance the HADS-A score. RP patients with high HADS-A score tended to belong to the Japan RP Society. The Japan RP society provides information about care and treatment to its members periodically, and they share a place to discuss the latest information on RP. This may be helpful for RP patients in reducing anxiety. Future investigations are necessary to determine whether patients with a strong anxiety join the RP society or whether the anxiety is lessened by joining the RP society.

The strength of this study was that it used the HADS-D method for psychological analysis which is accepted worldwide, and the questions were easy to answer by the patients. This made it convenient to collect comparable data from a large number of patients located at different hospitals. This is the first psychological analysis of Asian RP patients. Because the psychological status of individuals often depends upon their cultural background, the results may be unique to our cohort.

There are several limitations in this study. The study population was composed of older individuals with average age of 60.7 years. Thus, the depression might be due to ageing because older people tend to have more depression than younger individuals [30,31]. Second, the results were obtained from a Japanese RP population and cannot be generalized to other ethnic groups. Third, the psychological status of RP patients can be affected by many other factors such as doctors, family members, and the interval between the initial diagnosis of RP and the test times. Because our cases were collected from multiple hospitals, these data cannot be standardized. In addition, ocular pain, which is a factor that can affect the quality of life in general, was not included in this study because there have been earlier reports that no significant relationship was observed between NEI-VFQ25 score and ocular pain in RP patients [26, 27]. However, we cannot exclude the possibility that minimal ocular pain associated with RP may have affected the results.

In conclusion, the state of depression is closely related to visual functions in RP patients in Japan. For the successful care of RP patients with deteriorating vision, the state of depression should be monitored carefully. Attention should be paid to those who have other health problems or have difficulty in identifying their roles in their life.

## Supporting information

S1 Raw Data (XLSX)
